# Supplementary material for: A toolkit for studying cell surface shedding of diverse transmembrane receptors
Source: eLife. 2019 Jun 7;8:e46983. doi: 10.7554/eLife.46983 (PMC6586460; doi:10.7554/eLife.46983)
Supplement: Transparent reporting form [file elife-46983-transrepform.pdf]

eLIFE
1st Floor
24 Hills Road
Cambridge CB2 1JP, UK
P 01223 855340
W elifesciences.org
T @elife

eLife's transparent reporting form

We encourage authors to provide detailed information *within their submission* to facilitate the interpretation and replication of experiments. Authors can upload supporting documentation to indicate the use of appropriate reporting guidelines for health-related research (see EQUATOR Network), life science research (see the BioSharing Information Resource), or the ARRIVE guidelines for reporting work involving animal research. Where applicable, authors should refer to any relevant reporting standards documents in this form.

If you have any questions, please consult our Journal Policies and/or contact us: editorial@elifesciences.org.

Sample-size estimation

- You should state whether an appropriate sample size was computed when the study was being designed
- You should state the statistical method of sample size computation and any required assumptions
- If no explicit power analysis was used, you should describe how you decided what sample (replicate) size (number) to use

Please outline where this information can be found within the submission (e.g., sections or figure legends), or explain why this information doesn't apply to your submission:

Our submission does not include any population data or clinical study data, so sample-size estimation is not necessary.

Replicates

- You should report how often each experiment was performed
- You should include a definition of biological versus technical replication
- The data obtained should be provided and sufficient information should be provided to indicate the number of independent biological and/or technical replicates
- If you encountered any outliers, you should describe how these were handled
- Criteria for exclusion/inclusion of data should be clearly stated
- High-throughput sequence data should be uploaded before submission, with a private link for reviewers provided (these are available from both GEO and ArrayExpress)

Please outline where this information can be found within the submission (e.g., sections or figure legends), or explain why this information doesn't apply to your submission:

For ELISA Data (Figures 1D, Supplementary Figure 2, and Supplementary Figure 5C): Each experiment was performed at least twice with a biological replicate of three e.g., three wells of a 96 well plate were transfected with the same construct and read out separately on a plate reader.

For chimera signaling assay data (Figures 1C, 3B, 4B, Supplementary Figures 1,3,5): Notch was used as an internal control in every chimera experiment performed. All constructs except HER2 and HER4 were used in signaling assay at least twice using 10 ng DNA/well, at least once using 1 ng DNA/well, and at least once using 0.1 ng DNA/well. The HER2-Notch chimera was tested at least five times. The HER4-Notch

eLife Sciences Publications, Ltd is a limited liability non-profit non-stock corporation incorporated in the State of Delaware, USA, with company number 5030732, and is registered in the UK with company number FC030576 and branch number BR015634 at the address 1st Floor, 24 Hills Road, Cambridge CB2 1JP | August 2014

chimera was tested only once. For chimera signaling assay on plated ligand (Supplementary Figure 4, assay was performed 4 times in 96 well (2x) or 384 well plates (2x) with 10ng or 6.25ng DNA, respectively. Each signaling assay is performed with three biological replicates e.g., three wells of a 96 well plate were transfected with the same construct and read out separately on a plate reader.

For chimera assay with activated MMP (Figures 2B): Experiments performed with only activated MMP were performed three separate times with a biological replicate of three e.g., three wells of a 96 well plate were transfected with the same construct and read out separately on a plate reader.

For Western blot (Figure 4E): Western blots to compare MCF7 cell lysates treated with IgG control and DECMA-1 antibodies were performed as a biological replicate of three e.g., Cells were transfected and lysed on three separate dates. Each of the lysates was used for only one Western blot. Co-treatment of DECMA-1 and protease inhibitors was performed twice.

For Herceptin and DECMA-1 chimera assays (Figures 3C and 4C): Each signaling assay was performed 4 independent times over a range of antibody concentrations with biological replicates of three e.g., three wells of a 96 well plate were transfected with the same construct and read out separately on a plate reader.

Statistical reporting

- Statistical analysis methods should be described and justified
- Raw data should be presented in figures whenever informative to do so (typically when N per group is less than 10)
- For each experiment, you should identify the statistical tests used, exact values of N, definitions of center, methods of multiple test correction, and dispersion and precision measures (e.g., mean, median, SD, SEM, confidence intervals; and, for the major substantive results, a measure of effect size (e.g., Pearson's r, Cohen's d)
- Report exact p-values wherever possible alongside the summary statistics and 95% confidence intervals. These should be reported for all key questions and not only when the p-value is less than 0.05.

Please outline where this information can be found within the submission (e.g., sections or figure legends), or explain why this information doesn't apply to your submission:

All error bars in signaling and ELISA assays are reported as SEM. For Herceptin and DECMA-1 chimera signaling assays (Figures 3C and 4C), statistical information is given in the figure legend. ANOVA analysis was performed to assess statistical significance. We did not report p-values for the chimeric signaling assays in the text or figures. We instead report the p-values along with means and SEM for each chimera in a Source Data file linked to Figs 1 and 3

(For large datasets, or papers with a very large number of statistical tests, you may upload a single table file with tests, Ns, etc., with reference to sections in the manuscript.)

Group allocation

eLife Sciences Publications, Ltd is a limited liability non-profit non-stock corporation incorporated in the State of Delaware, USA, with company number 5030732, and is registered in the UK with company number FC030576 and branch number BR015634 at the address 1st Floor, 24 Hills Road, Cambridge CB2 1JP | August 2014

eLIFE
1st Floor
24 Hills Road
Cambridge CB2 1JP, UK
P 01223 855340
W elifesciences.org
T @elife

- Indicate how samples were allocated into experimental groups (in the case of clinical studies, please specify allocation to treatment method); if randomization was used, please also state if restricted randomization was applied
- Indicate if masking was used during group allocation, data collection and/or data analysis

Please outline where this information can be found within the submission (e.g., sections or figure legends), or explain why this information doesn't apply to your submission:

Our submission did not include any group allocation.

Additional data files ("source data")

- We encourage you to upload relevant additional data files, such as numerical data that are represented as a graph in a figure, or as a summary table
- Where provided, these should be in the most useful format, and they can be uploaded as "Source data" files linked to a main figure or table
- Include model definition files including the full list of parameters used
- Include code used for data analysis (e.g., R, MatLab)
- Avoid stating that data files are "available upon request"

Please indicate the figures or tables for which source data files have been provided:

Figure 1, Figure 3, Figure 4, Supplementary Fig 5 link to Source Data 1.

eLife Sciences Publications, Ltd is a limited liability non-profit non-stock corporation incorporated in the State of Delaware, USA, with company number 5030732, and is registered in the UK with company number FC030576 and branch number BR015634 at the address 1st Floor, 24 Hills Road, Cambridge CB2 1JP | August 2014
